# The State of the Art of Piezo1 Channels in Skeletal Muscle Regeneration

**DOI:** 10.3390/ijms23126616

**Published:** 2022-06-14

**Authors:** Annalisa Bernareggi, Alessandra Bosutti, Gabriele Massaria, Rashid Giniatullin, Tarja Malm, Marina Sciancalepore, Paola Lorenzon

**Affiliations:** 1 Department of Life Sciences, University of Trieste, 34127 Trieste, Italy; alessandra.bosutti@units.it (A.B.); gabriele.massaria@phd.units.it (G.M.); msciancalepore@units.it (M.S.); plorenzon@units.it (P.L.); 2 A.I. Virtanen Institute for Molecular Sciences, University of Eastern Finland, 70211 Kuopio, Finland; rashid.giniatullin@uef.fi (R.G.); tarja.malm@uef.fi (T.M.)

**Keywords:** Piezo1, myogenesis, Yoda1, myoblasts, satellite cells, myotubes, sarcopenia, muscle atrophy

## Abstract

Piezo1 channels are highly mechanically-activated cation channels that can sense and transduce the mechanical stimuli into physiological signals in different tissues including skeletal muscle. In this focused review, we summarize the emerging evidence of Piezo1 channel-mediated effects in the physiology of skeletal muscle, with a particular focus on the role of Piezo1 in controlling myogenic precursor activity and skeletal muscle regeneration and vascularization. The disclosed effects reported by pharmacological activation of Piezo1 channels with the selective agonist Yoda1 indicate a potential impact of Piezo1 channel activity in skeletal muscle regeneration, which is disrupted in various muscular pathological states. All findings reported so far agree with the idea that Piezo1 channels represent a novel, powerful molecular target to develop new therapeutic strategies for preventing or ameliorating skeletal muscle disorders characterized by an impairment of tissue regenerative potential.

## 1. Introduction

Mechanotransduction is defined by the ability of a cell to sense mechanical inputs and convert them into signalling events that elicit biological responses. The main molecular translators of this process are the mechanically-activated (MA) ion channels expressed in almost all tissues of the body [1].

The MA channels include a large family of ion channels able to respond to stretch, swelling, flow, and poking, for the proper growth, development, and health of cells and tissues. They are comprised of MA channels of large and of small conductance, the two-pore potassium channel (K2P) family that include TREK-1, TREK-2 and TRAAK, the ion channels OSCA/TMEM63, the Transient Receptor Potential (TRP) channels, and the Piezo1 and Piezo2 channels [1].

Skeletal muscle is a tissue heavily affected by mechanical strain that contributes to homeostasis. This suggests that the functional role of MA channels may be especially predominant in muscle compared to other non-contractile cell-types. Indeed, one of the first records of MA channel activity dates back to 1984 in chick skeletal muscle fibers [2]. Later, in 1990, studies conducted in dystrophic muscle cells underlined the first link between MA channel dysfunction and skeletal muscle pathology [3]. Since then, due to the better understanding of the variety of triggering stimuli, including, for instance, the stiffness of the substrate or pressure from expanding of surrounding tissues, MA channels have been reported in almost all types of cells, even extending to bacteria, fungi and plants, where they control physiological and pathological cellular responses [1]. Currently, it is also known that the functional role of MA channel activity is directed at detecting an excess of stress on the sarcolemma and to act as a “safety alarm” for the reinforcement of the cytoskeleton and for preventing cell lysis [4].

One of the most interesting aspects of the role of mechanical forces in skeletal muscle physiology is the impact on regeneration, which is a critical factor for maintaining proper motor function [5]. Skeletal muscle regeneration requires an adult *myogenesis*, which is controlled by the myogenic precursors derived from a cell population named muscle satellite cells (MuSCs). These cells are critically important for regeneration, and are located between the basal lamina and the sarcolemma of adult skeletal muscle fibers [6]. They are important not only for skeletal muscle growth after birth, but also for mediating regeneration after injury. When activated, MuSCs proliferate into myoblasts able to fuse with each other into multinucleated myotubes or to damaged fibers, in order to reconstitute fiber integrity and function. Therefore, MuSCs define the regenerative potential of skeletal muscle under healthy conditions. Unfortunately, the regenerative capacity of skeletal muscle tends to decline during aging [7,8] and in pathological conditions manifests as muscular dystrophies [9,10].

MuSCs are directly and continually exposed to mechanical signals resulting from the contractile activity of the skeletal muscle fibers. Indeed, mechanical stretch represents one of the critical stimuli for the activation of MuSCs. At the molecular level, MuSC stretching is coupled to an MA channel-mediated Ca^2+^ influx that most likely controls the functional state of these cells and promotes the release of many local factors, including myokines such as hepatocyte growth factor (HGF) and the gaseous transmitter nitric oxide (NO) [11,12], which regulates the vascular functions in the muscle. Most of the locally-released factors control activation, proliferation and differentiation of MuSCs in an autocrine/paracrine manner [13]. Importantly, the reduction in MA channel activity leads to an in vitro decline in the proliferation of MuSCs [12], which is in line with the reduction of MuSCs observed in intact fibers in response to mechanical unloading [14,15]. Moreover, the activation of MA channels has been reported to contribute to anabolic signalling during acute reloading following disuse atrophy [16], suggesting that MuSCs are key players to counteract skeletal muscle atrophy during aging and in conditions of immobilization and microgravity. The pattern and the intensity of the mechanical load differently affects the MuSC cell behavior: low and tonic stimulation prevents MuSC activation, whereas stronger stimulation activates these cells, leading to a hypertrophic phenotype of the skeletal muscle tissue [8]. The signalling cascades in the MuSCs, including IGF1/IL6/JAK/STAT3, that are activated during exercise, could be mediated by IGF1 and IL6 released from the myogenic and neighbouring cells [8]. Certainly, muscle tissue is highly vascularized and the MuSCs are in close proximity with endothelial cells, suggesting a potential interplay between the two cell-types [17].

Currently, largely due to growing interest in the function of Piezo channels, it is becoming clearer how MA channels sense and transduce mechanosensitive stimuli. However, the contribution of MA channel activity and the related signalling pathways involved in the pathophysiology of MuSCs remain largely unknown. One limitation is related to the controversial nature of the mechanical “transducers” that likely include a heterogeneous population of MA channels with different sensitivities to the type of mechanical stimulations, but also to the kinetics, expression and ion permeability of these molecular transducers. In addition, the identification of the specific role of each type of MA channel is strongly limited by the poor selectivity of the physical and chemical agonists/antagonists available to discriminate the different types of ion channels.

The Piezo channel family discovered by Coste et al. in 2010 [18] consists of the two isoforms, Piezo1 and Piezo2. Piezo1 is mainly present in tissues exposed to fluid pressure and flow such as erythrocytes, vascular endothelial cells, bladder urothelial cells, and chondrocytes. Piezo1 along with Piezo2 is also expressed in sensory neurons from trigeminal [19,20] and dorsal root ganglia (DRG) [18]. Both are permeable to cations, including calcium ions, but the Piezo1-mediated currents inactivate slower than the Piezo2 –mediated currents (18).

Interestingly, the synthetic molecule Yoda1 was found to activate Piezo1 channels without the application of any mechanical force [21]. Yoda1 binds to a narrow hydrophobic pocket of the Piezo1 channel protein and stabilizes the open conformation of the ion channel. In this way, Yoda1 increases the open channel probability and the cation flux across the pore [21,22]. Later, two other agonists, Jedi1 and Jedi2, were discovered [23]. Moreover, Dooku1 was shown to reversibly block Yoda1-evoked activation of Piezo1, leaving unaltered constitutive Piezo1 activity [24].

Given the specific hypersensitivity of the Piezo1 channel to mechanical forces, the availability of the first selective chemical agonist Yoda1 provides an intriguing therapeutic prospective, making this field of research very attractive [25].

Historically, the identification of Piezo1 channels in skeletal muscle cells began with a study performed on the C2C12 myogenic cell line [26]. A few years later, our group reported the functional expression of Piezo1 in primary myogenic precursors of mouse *Flexor Digitorum Brevis* (FDB) muscle fibers [27], and later, our observation was confirmed in the *Tibialis Anterior* (TA), *Extensor Digitorum Longus* and *Soleus* muscle fibers [28,29,30]. Taken as a whole, these studies suggested thePiezo1 channel as the main potential mechanical transducer in the control of the intracellular signaling required for the correct development, maintenance and regeneration of skeletal muscle tissue [31,32]

Structurally, Piezo1 forms large homotrimers in the cell membrane in which each subunit contains 2500 amino acids and about 24–40 transmembrane helices. The ion channel conducts Na^+^, K^+^, Mg^2+^ along with a relatively large Ca^2+^ permeability [18] which can play a specific signalling role in muscle precursors. Piezo1 senses a variety of mechanical stimuli, such as shear force, cell stretching, poking and stiffness [18]. These channels also detect the membrane tension derived from the presence of an asymmetric membrane distribution of negatively charged lipids [21] or from the presence of cholesterol [33]. The latter is consistent with a modulatory role of the lipid cell membrane composition (*force-from-lipid*; [34]). Moreover, Piezo1 is modulated by the cytoskeleton, and by cell membrane interactions with the extracellular matrix (*force-from-filament*; [34]).

The aim of this review is to summarize the evidence about the role of the Piezo1 in skeletal muscle tissue, primarily focusing on their role in controlling the regenerative capability of the myogenic precursors and on how the chemical activation of Piezo1 in these cells could bring a novel powerful pharmacological tool to counteract the effect of skeletal muscle aging, disuse and injuries. This idea is complimentary to the original concept by D. Beech and colleagues on the potential development of “exercise” pills acting via Piezo1 in muscle vasculature [25].

## 2. The Role of Piezo1 during Adult Myogenesis

### 2.1. Piezo1 in MuSC Activation

**Figure 1:**
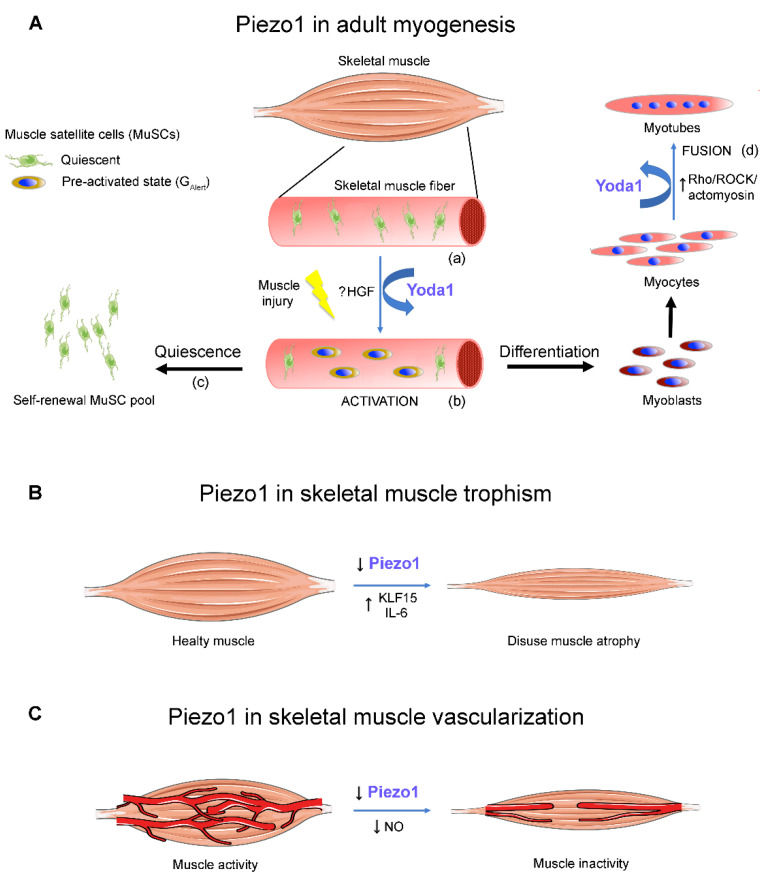
**Role of Piezo1 in skeletal muscle.** (**A**), Piezo1 controls the adult myogenesis. (**a**) Quiescent satellite cells (MuSCs) are located on intact muscle fibers. (**b**) Under muscle injury or following the Piezo1 activation by Yoda1, the MuSCs shift toward a G_Alert_ phase favouring the cell activation. (**c**) Part of the MuSCs self-renews the quiescent population; (**d**) the rest proliferates as myoblasts, differentiate into myocytes, which then fuse into multinucleated myotubes. The pharmacological activation of Piezo1 by Yoda1 promotes myocyte fusion and myotube differentiation. (**B**), A down regulation of PIEZO1 expression leads to disuse atrophy of the adult muscle. (**C**), The stimulation of the Piezo1 activity of endothelial cells and muscle cells during physical exercise sustain angiogenesis and muscle trophism.

The MuSCs are a heterogeneous population of cells coexisting in the same muscle fiber. Each step of the adult myogenesis is characterized by a time-specific expression of the transcription factor Pax7 and *Myogenic* *Regulatory *Factors** (MRFs) such as MyoD, Myf5, MyoG and Mrf4 [8]. The quiescent MuSC pool includes a long-term self-renewing *stem* cell population identified by the expression of Pax7^+^ but not Myf5 (Pax7^+^/Myf5^−^) and a *committed* short-term self-renewing population identified by the expression of Pax7 and Myf5 (Pax7^+^/Myf5^+^, Figure 1A(a) [8]).

During muscle injury, a subtype of committed MuSCs is recognizable, in a G_Alert_ reversible phase, and defines an ‘alerting’ pre-activated state of the cells. The G_Alert_ allows a quick activation under conditions of damage and stress, priming the MuSCs for cell cycle entry [35]. The G_Alert_ transition depends on systemic signals, and it is likely mediated by a downstream PI3K-Akt signalling, following the binding of HGF to the cMet receptor expressed by the MuSCs [35]. From a morphological point of view, the G_Alert_ phase is characterized by the lack of cell protrusions, a more circular cell shape and a larger size than its quiescent counterpart ([28], Figure 1A(b)).

According to the degree of muscle injury, the quiescent population Pax7^+^/Myf5^−^ can divide either symmetrically or asymmetrically. The symmetric division occurs to guarantee the self-renewal of the stem cell pool Pax7^+^/Myf5^−^; whereas the asymmetric division generates both the stem (Pax7^+^/Myf5^−^) and committed active (Pax7^+^/Myf5^+^) MuSCs. The correct balance between the two MuSC division modalities is fundamental to preserving the regenerative potential of the muscle: any imbalance reduces the pool of quiescent cells (Figure 1A(c)) as in age-related sarcopenia, or the pool of committed progenitors as in muscular dystrophies [36].

Once activated, the MuSCs give rise to a committed population of proliferating progenitor cells named *myoblasts* (Pax7^+^/Myf5^+^/MyoD^+^), which complete the terminal differentiation programme, becoming *myocytes* (MyoD^+^/MyoG^+^) which are ready to fuse into *myotubes* (MyoG^+^/Mrf4^+^; Figure 1A(d)).

Piezo1 is expressed early in quiescent Pax7^+^ MuSCs that are still attached to the muscle fibers [26,27,28,29]. The pharmacological activation of Piezo1 with Yoda1 does not affect the total number of Pax7^+^ cells both in in vivo or in in vitro conditions [27,28,29,30]. However, a significant reduction in the number of Pax7^+^ MuSCs has been reported after knocking down PIEZO1 gene-expression, suggesting that Piezo1 is required for preserving the pool of MuSCs [29,30].

Investigations on the role of Piezo1 following MuSC activation were initially being performed both on MuSCs in isolated muscle fibers [27,29,30] and in intact muscle [28]. Interestingly, in intact TA muscle, Ma and colleagues [28] analysed the morphological changes of MuSCs induced by activation of Piezo1 channels by Yoda1. They associated the functional MuSC activity to the number and length of cell protrusions able to sense the microenvironment and identified three different MuSC subtypes; “responsive” (small and rounded, with 0–1 axon-like cytoplasmic extensions, less than 40% of the total MuSCs), “sensory” (large, with cytoplasmic extensions, less than 5%), and “intermediate” (with morphological properties in between the above subtypes, about 50%). Notably, after the in vivo administration of Yoda1, the cell morphology shifted toward the “responsive” phenotype; in the presence of the Piezo1 agonist there was a prevalence of the circular phenotype of MuSCs, similar of the *committed* cells in the G_Alert_ functional phase (Figure 1A(b)). Interestingly, the shift was reported in the absence of any regenerative signal, suggesting that Piezo1 activity favours the regenerative potential of MuSCs. This hypothesis was supported by the experiments in which, after PIEZO1 deletion, a change in cell morphology versus a more quiescent “sensory” phenotype was observed [28].

In another study, Peng and co-workers [29] investigated the role of Piezo1 in isolated TA muscle fibers. They reported an immunofluorescence-detected decline in the PIEZO1 expression in the Pax7^+^/MyoD^+^ MuSC population and that Yoda1 treatment reduced the number of the Pax7^+^/MyoD^+^ without affecting the total number of Pax7^+^ cells. On the contrary, the lack of PIEZO1 expression caused a reduction of the total number of Pax7^+^ cells and a relative increase in those expressing Pax7^+^/MyoD^+^, demonstrating a tendency towards a faster cell activation in the absence of Piezo1 activity. These findings indicate that Piezo1 secures a long-term MuSC quiescence pool and suggest that Piezo1 activity is needed to preserve the regenerative capability of the skeletal muscle.

### 2.2. Piezo1 in Myoblast Fusion

It has been recently shown that the expression of Piezo1 tends to increase during in vitro myogenesis [29]. The presence of Piezo1 in myocytes was first reported by Tsuchiya et al. [26] in the C2C12 cell line and then confirmed by our group and others in myocytes derived from freshly isolated FDB fibers [27]. With this model of primary cells, the pharmacological stimulation of Piezo1 with Yoda1 increased the fusion index, suggesting the ability of Piezo1 to promote myoblast fusion ([27]; Figure 1A(c)). Such a role in cell fusion, which is a critical step for muscle development and regeneration, was further confirmed by the decline in the fusion index observed, both in vivo and in vitro, after silencing of PIEZO1 [29,30].

Our understanding of the entire machinery involved in this process is still largely incomplete, but the role of the mechanical tension is important [37]. During fusion, the cell membrane undergoes several changes that affect the plasmalemma tension, and Piezo1 is activated due to cytoskeletal tethers (*force from filament*) and membrane tension (*force from lipids*) [38].

Moreover, since Piezo1 activity is affected by the lipidic composition of the cell membrane [39], a correct cell surface flip-flop of phosphatidylserine was found to control the myoblast fusion by regulating Piezo1 activity via a *force from lipids* effect [26]. In line with this, the increased exposure of phosphatidylserine induced by TMEM16F scramblase stimulates the fusion of the C2C12 cells [40], whereas the prevalence of phosphatidylserine inner translocation mediated by P4-ATP-ase flippase avoids the excess of syncytia formation [26].

Piezo1 activity controls the Ca^2+^ influx across the cell membrane needed for the cortical actinomyosin assembly via the Rho/ROCK/actomyosin pathway, which is also crucial for myotube formation; the silencing of PIEZO1 leads to an excessive cell fusion and elongation defects [26]. Notably, the pharmacological activation with Yoda1 improves the orientation of the cells in favour of a correct fusion and myotube formation [27,30].

Intriguingly, the type of mechanical stress (acute or persistent), its magnitude, as well as the subcellular localization of applied forces [41] might activate specific intracellular signalling [42] and thus induce different physiological responses. For instance, in cardiomyocytes, Piezo1 activation by mechanical stimuli elicits an intracellular Ca^2+^ increase, promoting a Ca^2+^ influx, or a Ca^2+^ release from intracellular stores controlling ROS production [43]. Interestingly, in skeletal muscle, low levels of intracellular ROS favour myoblast proliferation/differentiation [44], stimulate CREB (cAMP response element-binding protein) mediated gene expression in myotubes as well as the downstream ERK1/2, promoting muscle regeneration [45]. On the other hand, Piezo1 activated by destructive mechanical forces might trigger inflammatory cascades resulting in immune cell infiltration, the activation of pro-inflammatory agents such as IL-6 and large ROS production that contribute to the development of degenerative diseases and pathological conditions [46]. The latter observations indicate that the negative modulation of Piezo1 could reduce the inflammation and represent a potentially helpful therapeutic tool.

In skeletal myotubes, Piezo1 is also important for the maintenance of basal [Ca^2+^]_i_, even in the absence of any induced mechanical stimulation. This mechanism appears to be important for the down-regulation of atrophy-related genes [47]. Intriguingly, since before synaptogenesis, spontaneous electrical and mechanical activity detected in differentiating myotubes can regulate the Ca^2+^ homeostasis [48,49], Piezo1 could contribute to the control of the level of [Ca^2+^]_i_ to sustain the trophism of the myotubes before the arrival of the nerve [27].

### 2.3. Piezo1 in Adult Skeletal Muscle Fibers

While the role of the Piezo1 activity during the early steps of myogenesis begins to be unveiled, there are still only a few studies carried out on adult muscle fibers. PIEZO1 expression was first reported using RT-PCR analysis in murine adult muscle fibers [26]. More recently, we reported the existence of Piezo1 clusters in isolated mouse FDB muscle fibers, characterized by a smaller size than in myotubes [27]. After that, Piezo1 was also found in mouse *Gastrocnemius* muscle fibers [47]. Notably, the expression of Piezo1 has been found to decline in disused atrophic muscle (Figure 1B) [47]. In addition, it has also been shown that the decline in the expression and the consequent reduction in Piezo1 activity lead to an up-regulation of atrophy-related genes [47]. The decrease in PIEZO1 expression in response to disuse was also confirmed by the analysis of human muscle biopsies derived from patients who had undergone cast fixation upon bone fracture [47]. All of these findings strongly support the hypothesis that Piezo1 is involved in the control of skeletal muscle trophism (Figure 1B).

In isolated mouse FDB muscle fibers, the chemical activation of Piezo1 with Yoda1 failed to elicit a detectable variation of the [Ca^2+^]_i_ [27]. However, the intramuscular injection of Yoda1 increased the [Ca^2+^]_i_ in intact mouse TA muscle, and down regulated the expression of the atrophy-related genes Kruppel-like factor 15 (KLF15) and IL-6 [47]. The apparent discrepancy in these findings may be due to the different cell models analysed and, in particular, to a different sensitivity of Piezo1 to the chemical activation in isolated skeletal muscle fibers, in which the plasmalemma tension forces are likely different than in the intact whole muscle.

## 3. Possible Role of Piezo1 in the Cooperation between Vascular and Skeletal Muscle Cell Networks

One interesting aspect of Piezo 1 activity in skeletal muscle is related to the cooperation between vascular and skeletal muscle cells. Prolonged periods of muscle inactivity, like bed rest, space flight, neuromuscular diseases or aging, cause arterial structural remodeling and reduced blood flow to the muscle [50] (Figure 1C).

Successful angiogenesis and capillary maintenance critically depend on mechanical signals and endothelial cell shear stress, which can involve Piezo1 activation. Piezo1 expressed in endothelial cells acts as a sensor to control blood flow in skeletal muscles during exercise [51,52]. In saphenous arteries, the activation of endothelial Piezo1 triggers a Ca^2+^ influx likely responsible for NO production, which promotes vasodilatation [52] and therefore supply to muscle (Figure 1C). Interestingly, NO also stimulates the activation of MuSCs promoting muscle regeneration [53]. Moreover, the normal shear between the sarcolemma and basal lamina has been reported to facilitate a pulsatile release of NO from the myofibers and likely from MuSCs [53].

On the other hand, capillary rarefaction and impaired muscle oxygenation contribute to the progressive loss in the ability to exercise and resistance to fatigue [54,55,56]. MuSCs are localized close to vessels and their number is positively correlated to the fiber capillarization, suggesting a MuSC-vessel cross-talk [17,57]. The basal lamina, the pericytes, and the smooth muscles apparently prevent any direct contact between MuSCs and endothelial cells. However, there is evidence of gaps in the basal lamina, which allows the communication between these two cell types [58]. Moreover, the remodeling of the tissue during muscle regeneration increases the distance between pericytes and capillaries. This likely further favours the interaction between MuSCs and endothelial cells, promoting angiogenesis and myogenesis [59].

Even if premature, we could speculate a simultaneous synergism of Piezo1-mediated NO release from endothelial cells, adult muscle fibers and MuSCs that improves the muscle performance. If this turns out to be true, Piezo1 could control the local NO levels (and maybe other released factors) contributing to the cross-talk between skeletal muscle tissue and vessels. This hypothesis could well explain the positive correlation between myofiber size and its number of capillaries [60,61]

A MuSC-endothelial cell cross-talk is supported, via paracrine mechanisms, by the released factors: MuSCs release VEGFA (vascular endothelial growth factor A), promoting angiogenesis and capillarization of muscle tissue [57] and endothelial cells release IGF-1, HGF and bFGF stimulating the myogenic cell growth [58].

The mutual dependence between capillary bed and MuSCs indicates that the angiogenesis might affect myogenesis and *vice-versa* [59]. Therefore, a functional capillary supply would be not only essential to delivering oxygen and nutrients to muscle tissue and for removal of heat, metabolites, and waste products but also to positively modulate the growth and regenerative capacity of muscles [54]. In line with this, capillary rarefaction in prolonged inactivity has a negative effect on muscle remodeling and repair [57].

## 4. Translational Perspectives for Developing Piezo1-Based Therapeutic Strategies

### 4.1. Skeletal Muscle Pathologies and Aging

As discussed in this review, Piezo1 is a mechanical transducer that controls both maintenance and activation of the MuSC pool as well as differentiation of myotubes. From these findings, Piezo1 emerges as a potential new molecular target for developing new treatments of various muscle pathologies associated with impaired adult myogenesis [36].

The pharmacological activation of Piezo1 partially restores the defective morphology of the dystrophic MuSCs [28]. Moreover, the observations that the Yoda1 treatment likely contributes to the Ca^2+^ influx promoting cell fusion [27,30] and differentiation [27,30] suggest that Piezo1 could also be an important tool for the treatment of cell-cell fusion deficits reported in some genetic myopathies [62].

The regenerative capability of skeletal muscle also decreases during aging [7,8]. Sarcopenia is a common aged-related chronic condition characterized by loss of muscle mass (hypotrophy), strength and/or physical function impairment, leading to an increased risk of disability and to a poor quality of life in the elderly [63]. During aging, the increase of fibroses, as well the stiffness of the extracellular matrix, affect the mechanical sensitivity of MuSCs and cause defects in the expression of myogenic growth factors and protein synthesis of the skeletal muscle fibers [64].

The recent discovery that an impaired Piezo1 activity in MuSCs increases ROS production and accumulation of p53 tumor suppressor protein stimulating a massive induction of cell senescence phenomena [29] strongly suggest the crucial role of Piezo1 in the physiopathology of skeletal muscle aging [65]. The repetitive passive stretching of the sarcopenic muscle fibers recovers the hypotrophy and restores the expression of MRFs in MuSCs [5]. Appropriate physical exercises combined with a pharmacological activation of Piezo1 could represent a promising approach to counteract more efficiently the sarcopenic status and the decline of muscle trophism in the elderly.

### 4.2. Skeletal Muscle Atrophy

The presence of Piezo1 has been reported in adult skeletal muscle fibers, even though the downstream pathways are unknown, and deserves to be investigated in future studies. Some lessons could be taken from mature cardiomyocytes, where Piezo1 channels are important mechanosensors in mature cardiomyocytes, and they are localized near the T-tubules and intercalated discs. In cardiac cells, the Piezo1 activity does not contribute much to the basal Ca^2+^ level, but upon mechanical or chemical stimulation, it elicits Ca^2+^-mediated calpain and calcineurin activation, responsible for the cardiac hypertrophy [66].

Calpain and calcineurin are also differently involved in skeletal muscle remodeling in response to exercise and inactivity-induced muscle atrophy [67,68]. In addition, in skeletal muscle fibers after immobilization or denervation, a reduction in the [Ca^2+^]_i_ associated with a down regulation of PIEZO1 expression and an up regulation of atrophy-related genes *Klfl5* and *Il6* expression has been reported [47] (Figure 1B). The intramuscular administration of Yoda1 suppresses the expression of atrophy-related genes and restores the [Ca^2+^]_i_ [47], suggesting that the modulation of Piezo1 activity could be used to prevent skeletal muscle atrophy.

### 4.3. Skeletal Muscle Adaptation to Microgravity

Microgravity induces substantial cellular adaptations that affect cellular morphology, proliferation and adhesion [69]. Cell mechanical properties, including elasticity, viscoelasticity [70], and cytoskeletal organization (namely mechano-responsive structures) are the most affected cell compartments in real or simulated microgravity [71]. Microgravity disrupts the intracellular tension balance and causes irregular cytoskeleton formation, with substantial implications in the regulation of cell growth, locomotion and survival [72]. For example, extracellular matrix proteins, cytoskeletal components and intermediate filaments are significantly affected in human endothelial cells when cultured in a random positioning machine simulating microgravity conditions [73]. Since the mechanics of the microenvironment can modulate Piezo1 signalling [74], it is likely that similar adaptations can occur at the skeletal muscle level, in its adult cell compartments and/or in stem cell niches. On this basis, microgravity and consequent mechanical unloading could affect MuSC membrane stiffness, leading to perturbations in Piezo1 activation, with a consequent impairment in the myogenesis signalling cascade [75]. This would, at least in part, explain why some markers of myogenesis (MyoG, Mylpf, and Myh3) appear downregulated in mouse skeletal C2C12 myoblasts when cultured in simulated microgravity conditions [76].

The possibility to mechanically or chemically tune the Piezo1 activity could be used to counteract the negative effects of microgravity during prolonged space flights and in the long-lasting stay in extra-terrestrial habitats.

### 4.4. Dietary Strategies to Control Piezo1 Activity?

**Figure 2:**
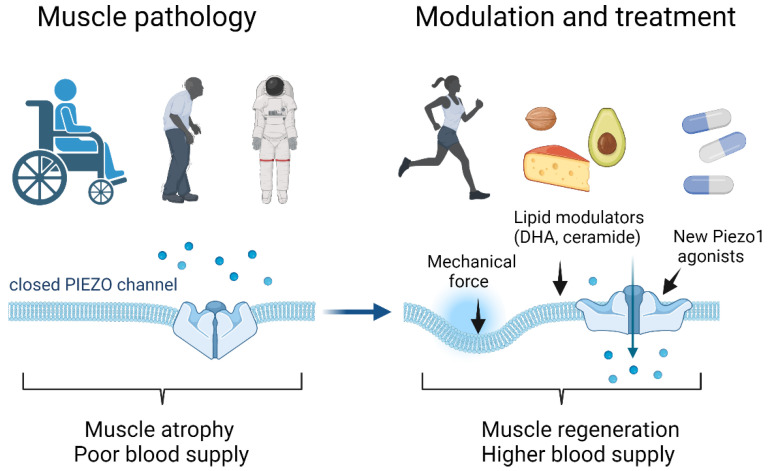
**Potential Piezo1-based therapeutic strategies to counteract skeletal muscle impairments.** Muscle disuse, aging and unloading might reduce Piezo1 activity, contributing to muscle atrophy and poor blood supply. Potentiation of Piezo 1 activity by physical exercise, diet and chemical/pharmacological activation might enhance the regenerative potential and, possibly, skeletal muscle vascularization and provide a higher blood supply. DHA and ceramide are lipids which are able to enhance the activity of Piezo1 channels, reducing their limiting inactivation state. These indirect and more direct Piezo1-based therapeutic strategies based on novel mechanisms of Piezo1 activation could thus represent promising new countermeasures versus atrophy and diseases related to muscle weaknesses (Figure created with Biorender.com, accessed on 2 May 2022).

Due to the potential implication of Piezo1 in cardiovascular and inflammatory conditions [43,46,77], increasing attention has been directed in the last years to characterize new potential treatments capable of pharmacologically modulating Piezo1 function, or acting on the elastic properties of the plasma membrane, such as its lipid/cholesterol components, which regulate Piezo1 function in a dynamic manner [39]. Some in vitro studies underline statins, methyl-β-cyclodextrin, or dynasore (a GTPase inhibitor) and dietary fatty acid supplementation, in modulating Piezo 1 activation/inhibition by acting on the membrane cholesterol content and fatty acid metabolism [33,34,78,79]. In this context, in an elegant study, Romero and colleagues [34] have demonstrated that the cellular intrinsic lipid profile and changes in fatty acid metabolism are capable of remodeling the cellular response to mechanical stimuli. Fatty acid diet-supplementation can abrogate the phenotype of “gain-of-function Piezo1 mutations”, which is linked to stomatocytosis, a human hereditary disease. Interestingly, as shown by these authors, non-saturated fatty acids may have an opposite effect on Piezo1 channels. In particular, the action of docosahexaenoic acid (DHA), contrary to that of eicosapentaenoic acid (EPA), is associated with the reduced inactivation of Piezo1, which might be translated as a promising effect to increase the channel activity in skeletal muscle (Figure 2). Likewise, ceramide, produced by sphingomyelin phosphodiesterase 3 [80] (Figure 2), is a powerful tool to remove inactivation of Piezo1. While the former can be widely used in patients with sarcopenia, the latter requires more exploration to be used as a therapeutic tool.

## 5. Conclusions

There is growing evidence that Piezo1 controls trophism and the regenerative capability of skeletal muscle cells by transducing mechanical stimuli. According to this view, Piezo1 can be considered a physiological key player in skeletal muscle remodeling, tissue repair and perhaps in muscle vascularization. Physical exercise is well documented to attenuate some disuse-induced deficiency in skeletal muscle function, and this may be at least partially mediated through Piezo1 activation. Nonetheless, exercise alone does not fully protect the skeletal muscle system from prolonged periods of disuse, and there is an urgent need to develop a means of fully counteracting the effects of disuse. Pharmacological enhancement of Piezo1 activity by combining physical exercise with the diet then emerges as a potentially new powerful strategy for protecting skeletal muscle from the consequences of aging and skeletal muscle pathologies (Figure 2).

## Data Availability

Not applicable (no data reported in this review).

## Abbreviations

MuSCs	Muscle Satellite Cells
MA channels	Mechanically-activated ion channels
MyoG	Myogenin
NO	Nitric Oxide
IGF	Insulin Growth Factor
MRF	Myogenic Regulatory factor
TA	Tibialis Anterior
FDB	Flexor Digitorum Brevis

## References

[1] Kefauver J.M., Ward A.B., Patapoutian A. (2020). Discoveries in structure and physiology of mechanically activated ion channels. Nature.

[2] Guharay F., Sachs F. (1984). Stretch-activated single ion channel currents in tissue-cultured embryonic chick skeletal muscle. J. Physiol..

[3] Franco A., Lansman J.B. (1990). Calcium entry through stretch-inactivated ion channels in mdx myotubes. Nature.

[4] Huang H., Bae C., Sachs F., Suchyna T.M. (2013). Caveolae regulation of mechanosensitive channel function in myotubes. PLoS ONE.

[5] Wang Y., Song J., Liu X., Liu J., Zhang Q., Yan X., Yuan X., Ren D. (2020). Multiple Effects of Mechanical Stretch on Myogenic Progenitor Cells. Stem Cells Dev..

[6] Mauro A. (1961). Satellite cell of skeletal muscle fibers. J. Biophys. Biochem. Cytol..

[7] Collins C.A., Zammit P.S., Ruiz A.P., Morgan J.E., Partridge T.A. (2007). A population of myogenic stem cells that survives skeletal muscle aging. Stem Cells.

[8] Chen W., Datzkiw D., Rudnicki M.A. (2020). Satellite cells in ageing: Use it or lose it. Open Biol..

[9] Blau H.M., Webster C., Pavlath G.K. (1983). Defective myoblasts identified in Duchenne muscular dystrophy. Proc. Natl. Acad. Sci. USA.

[10] Chang N.C., Chevalier F.P., Rudnicki M.A. (2016). Satellite Cells in Muscular Dystrophy—Lost in Polarity. Trends Mol. Med..

[11] Tatsumi R., Sheehan S.M., Iwasaki H., Hattori A., Allen R.E. (2001). Mechanical stretch induces activation of skeletal muscle satellite cells in vitro. Exp. Cell Res..

[12] Hara M., Tabata K., Suzuki T., Do M.K., Mizunoya W., Nakamura M., Nishimura S., Tabata S., Ikeuchi Y., Sunagawa K. (2012). Calcium influx through a possible coupling of cation channels impacts skeletal muscle satellite cell activation in response to mechanical stretch. Am. J. Physiol. Cell Physiol..

[13] Yin H., Price F., Rudnicki M.A. (2013). Satellite cells and the muscle stem cell niche. Physiol. Rev..

[14] Babcock L.W., Knoblauch M., Clarke M.S. (2015). The role of myostatin and activin receptor IIB in the regulation of unloading-induced myofiber type-specific skeletal muscle atrophy. J. Appl. Physiol..

[15] Arentson-Lantz E.J., English K.L., Paddon-Jones D., Fry C.S. (2016). Fourteen days of bed rest induces a decline in satellite cell content and robust atrophy of skeletal muscle fibers in middle-aged adults. J. Appl. Physiol..

[16] Mirzoev T.M., Tyganov S.A., Petrova I.O., Shenkman B.S. (2019). Acute recovery from disuse atrophy: The role of stretch-activated ion channels in the activation of anabolic signaling in skeletal muscle. Am. J. Physiol.-Endocrinol. Metab..

[17] Verma M., Asakura Y., Murakonda B.S.R., Pengo T., Latroche C., Chazaud B., McLoon L.K., Asakura A. (2018). Muscle Satellite Cell Cross-Talk with a Vascular Niche Maintains Quiescence via VEGF and Notch Signaling. Cell Stem Cell.

[18] Coste B., Mathur J., Schmidt M., Earley T.J., Ranade S., Petrus M.J., Dubin A.E., Patapoutian A. (2010). Piezo1 and Piezo2 are essential components of distinct mechanically activated cation channels. Science.

[19] Mikhailov N., Leskinen J., Fagerlund I., Poguzhelskaya E., Giniatullina R., Gafurov O., Malm T., Karjalainen T., Gröhn O., Giniatullin R. (2019). Mechanosensitive meningeal nociception via Piezo channels: Implications for pulsatile pain in migraine?. Neuropharmacology.

[20] Mikhailov N., Plotnikova L., Singh P., Giniatullin R., Hämäläinen R.H. (2022). Functional Characterization of Mechanosensitive Piezo1 Channels in Trigeminal and Somatic Nerves in a Neuron-on-Chip Model. Int. J. Mol. Sci..

[21] Syeda R., Xu J., Dubin A.E., Coste B., Mathur J., Huynh T., Matzen J., Lao J., Tully D.C., Engels I.H. (2015). Chemical activation of the mechanotransduction channel Piezo1. Elife.

[22] Lacroix J.J., Botello-Smith W.M., Luo Y. (2018). Probing the gating mechanism of the mechanosensitive channel Piezo1 with the small molecule Yoda. Nat. Commun..

[23] Wang Y., Chi S., Guo H., Li G., Wang L., Zhao Q., Rao Y., Zu L., He W., Xiao B. (2018). A lever-like transduction pathway for long-distance chemical- and mechano-gating of the mechanosensitive Piezo1 channel. Nat. Commun..

[24] Evans E.L., Cuthbertson K., Endesh N., Rode B., Blythe N.M., Hyman A.J., Hall S.J., Gaunt H.J., Ludlow M.J., Foster R. (2018). Yoda1 analogue (Dooku1) which antagonizes Yoda1-evoked activation of Piezo1 and aortic relaxation. Br. J. Pharmacol..

[25] Beech D.J. (2018). Endothelial Piezo1 channels as sensors of exercise. J. Physiol..

[26] Tsuchiya M., Hara Y., Okuda M., Itoh K., Nishioka R., Shiomi A., Nagao K., Mori M., Mori Y., Ikenouchi J. (2018). Cell surface flip-flop of phosphatidylserine is critical for PIEZO1-mediated myotube formation. Nat. Commun..

[27] Bosutti A., Giniatullin A., Odnoshivkina Y., Giudice L., Malm T., Sciancalepore M., Giniatullin R., D’Andrea P., Lorenzon P., Bernareggi A. (2021). “Time window” effect of Yoda1-evoked Piezo1 channel activity during mouse skeletal muscle differentiation. Acta Physiol..

[28] Ma N., Chen D., Lee J.H., Kuri P., Hernandez E.B., Kocan J., Mahmood H., Tichy E.D., Rompolas P., Mourkioti F. (2022). Piezo1 regulates the regenerative capacity of skeletal muscles via orchestration of stem cell morphological states. Sci. Adv..

[29] Peng Y., Du J., Günther S., Guo X., Wang S., Schneider A., Zhu L., Braun T. (2022). Mechano-signaling via Piezo1 prevents activation and p53-mediated senescence of muscle stem cells. Redox Biol..

[30] Ortuste Quiroga H.P., Ganassi M., Yokoyama S., Nakamura K., Yamashita T., Raimbach D., Hagiwara A., Harrington O., Breach-Teji J., Asakura A. (2022). Fine-Tuning of Piezo1 Expression and Activity Ensures Efficient Myoblast Fusion during Skeletal Myogenesis. Cells.

[31] Wang M.J., Zhu Y.C., Shi J. (2021). A crucial physiological role of Piezo1 channel in differentiation rather than proliferation during myogenesis. Acta Physiol..

[32] Jagasia R., Wagner K.R. (2022). Piezo1: Opening the way to preventing muscle atrophy. J. Clin. Investig..

[33] Ridone P., Pandzic E., Vassalli M., Cox C.D., Macmillan A., Gottlieb P.A., Martinac B. (2020). Disruption of membrane cholesterol organization impairs the activity of PIEZO1 channel clusters. J. Gen. Physiol..

[34] Romero L.O., Massey A.E., Mata-Daboin A.D., Sierra-Valdez F.J., Chauhan S.C., Cordero-Morales J.F., Vásquez V. (2019). Dietary fatty acids fine-tune Piezo1 mechanical response. Nat. Commun..

[35] Rodgers J.T., King K.Y., Brett J.O., Cromie M.J., Charville G.W., Maguire K.K., Brunson C., Mastey N., Liu L., Tsai C.R. (2014). mTORC1 controls the adaptive transition of quiescent stem cells from G0 to G(Alert). Nature.

[36] Feige P., Brun C.E., Ritso M., Rudnicki M.A. (2018). Orienting Muscle Stem Cells for Regeneration in Homeostasis, Aging, and Disease. Cell Stem Cell.

[37] Kim J.H., Jin P., Duan R., Chen E.H. (2015). Mechanisms of myoblast fusion during muscle development. Curr. Opin. Genet. Dev..

[38] Guo Y.R., MacKinnon R. (2017). Structure-based membrane dome mechanism for Piezo mechanosensitivity. Elife.

[39] Yang X., Lin C., Chen X., Li S., Li X., Xiao B. (2022). Structure deformation and curvature sensing of PIEZO1 in lipid membranes. Nature.

[40] Zhao P., Torcaso A., Mariano A., Xu L., Mohsin S., Zhao L., Han R. (2014). Anoctamin 6 regulates C2C12 myoblast proliferation. PLoS ONE.

[41] Gudipaty S.A., Lindblom J., Loftus P.D., Redd M.J., Edes K., Davey C.F., Krishnegowda V., Rosenblatt J. (2017). Mechanical stretch triggers rapid epithelial cell division through Piezo1. Nature.

[42] Ingber D.E. (1997). Tensegrity: The architectural basis of cellular mechanotransduction. Annu. Rev. Physiol..

[43] Jiang F., Yin K., Wu K., Zhang M., Wang S., Cheng H., Zhou Z., Xiao B. (2021). The mechanosensitive Piezo1 channel mediates heart mechano-chemo transduction. Nat. Commun..

[44] Sciancalepore M., Luin E., Parato G., Ren E., Giniatullin R., Fabbretti E., Lorenzon P. (2012). Reactive oxygen species contribute to the promotion of the ATP-mediated proliferation of mouse skeletal myoblasts. Free Radic. Biol. Med..

[45] Espinosa A., Leiva A., Pena M., Muller M., Debandi A., Hidalgo C. (2006). Myotube depolarization generates reactive oxygen species through NAD(P)H oxidase; ROS-elicited Ca2+ stimulates ERK, CREB, early genes. J. Cell. Physiol..

[46] Liu H., Hu J., Zheng Q., Feng X., Zhan F., Wang X., Xu G., Hua F. (2022). Piezo1 Channels as Force Sensors in Mechanical Force-Related Chronic Inflammation. Front. Immunol..

[47] Hirata Y., Nomura K., Kato D., Tachibana Y., Niikura T., Uchiyama K., Hosooka T., Fukui T., Oe K., Kuroda R. (2022). A Piezo1/KLF15/IL-6 axis mediates immobilization-induced muscle atrophy. J. Clin. Investig..

[48] Bandi E., Bernareggi A., Grandolfo M., Mozzetta C., Augusti-Tocco G., Ruzzier F., Lorenzon P. (2005). Autocrine activation of nicotinic acetylcholine receptors contributes to Ca2+ spikes in mouse myotubes during myogenesis. J. Physiol..

[49] Sciancalepore M., Afzalov R., Buzzin V., Jurdana M., Lorenzon P., Ruzzier F. (2005). Intrinsic ionic conductances mediate the spontaneous electrical activity of cultured mouse myotubes. Biochim. Biophys. Acta-Biomembr..

[50] Thijssen D.H., Green D.J., Hopman M.T. (2011). Blood vessel remodeling and physical inactivity in humans. J. Appl. Physiol..

[51] Rode B., Shi J., Endesh N., Drinkhill M.J., Webster P.J., Lotteau S.J., Bailey M.A., Yuldasheva N.Y., Ludlow M.J., Cubbon R.M. (2017). Piezo1 channels sense whole body physical activity to reset cardiovascular homeostasis and enhance performance. Nat. Commun..

[52] Bartoli F., Debant M., Chuntharpursat-Bon E., Evans E.L., Musialowski K.E., Parsonage G., Morley L.C., Futers T.S., Sukumar P., Bowen T.S. (2022). Endothelial Piezo1 sustains muscle capillary density and contributes to physical activity. J. Clin. Investig..

[53] Anderson J.E. (2000). A role for nitric oxide in muscle repair: Nitric oxide-mediated activation of muscle satellite cells. Mol. Biol. Cell.

[54] Bosutti A., Salanova M., Blottner D., Buehlmeier J., Mulder E., Rittweger J., Yap M.H., Ganse B., Degens H. (2016). Whey protein with potassium bicarbonate supplement attenuates the reduction in muscle oxidative capacity during 19 days of bed rest. J. Appl. Physiol..

[55] Blottner D., Hastermann M., Weber R., Lenz R., Gambara G., Limper U., Rittweger J., Bosutti A., Degens H., Salanova M. (2020). Reactive Jumps Preserve Skeletal Muscle Structure, Phenotype, and Myofiber Oxidative Capacity in Bed Rest. Front. Physiol..

[56] Tickle P.G.P., Hendrickse W., Degens H., Egginton S. (2020). Impaired skeletal muscle performance as a consequence of random functional capillary rarefaction can be restored with overload-dependent angiogenesis. J. Physiol..

[57] Collins B.C., Kardon G. (2018). Won’t You Be My Neighbor? Muscle Stem Cells Recruit Endothelial Cells to Their Niche. Cell Stem Cell.

[58] Latroche C., Gitiaux C., Chrétien F., Desguerre I., Mounie R., Chazaud B. (2015). Skeletal muscle microvasculature: A highly dynamic lifeline. Physiology.

[59] Christov C., Chrétien F., Abou-Khalil R., Bassez G., Vallet G., Authier F.J., Bassaglia Y., Shinin V., Tajbakhsh S., Chazaud B. (2007). Muscle satellite cells and endothelial cells: Close neighbors and privileged partners. Mol. Biol. Cell.

[60] Wüst R.C.I., Morse C.I., Haan A., Rittweger J., Jones D.A., Degens H. (2008). Skeletal muscle properties and fatigue resistance in relation to smoking history. Eur. J. Appl. Physiol..

[61] Bosutti A., Egginton S., Barnouin Y., Ganse B., Rittweger J.R., Degens H. (2015). Local capillary supply in muscle is not determined by local xidative capacity. J. Exp. Biol..

[62] Di Gioia S.A., Connors S., Matsunami N., Cannavino J., Rose M.F., Gilette N.M., Artoni P., de Macena Sobreira N.L., Chan W.M., Webb B.D. (2017). A defect in myoblast fusion underlies Carey-Fineman-Ziter syndrome. Nat. Commun..

[63] Cruz-Jentoft A.J., Sayer A.A. (2019). Sarcopenia. Lancet.

[64] Lacraz G., Rouleau A.J., Couture V., Söllrald T., Drouin G., Veillette N., Grandbois M., Grenier G. (2015). Increased Stiffness in Aged Skeletal Muscle Impairs Muscle Progenitor Cell Proliferative Activity. PLoS ONE.

[65] Boers H.E., Haroon M., Le Grand F., Bakker A.D., Klein-Nulend J., Jaspers R.T. (2018). Mechanosensitivity of aged muscle stem cells. J. Orthop. Res..

[66] Zhang Y., Su S.A., Li W., Ma Y., Shen J., Wang Y., Shen Y., Chen J., Ji Y., Xie Y. (2021). Piezo1-Mediated Mechanotransduction Promotes Cardiac Hypertrophy by Impairing Calcium Homeostasis to Activate Calpain/Calcineurin Signaling. Hypertension.

[67] Hyatt H.W., Powers S.K. (2020). The Role of Calpains in Skeletal Muscle Remodeling with Exercise and Inactivity-induced Atrophy. Int. J. Sports Med..

[68] Sakuma K., Yamaguchi A. (2010). The functional role of calcineurin in hypertrophy, regeneration, and disorders of skeletal muscle. J. Biomed. Biotechnol..

[69] Bradbury P., Wu H., Choi J.U., Rowan A.E., Zhang H., Poole K., Lauko J., Chou J. (2020). Modeling the Impact of Microgravity at the Cellular Level: Implications for Human Disease. Front. Cell. Dev. Biol..

[70] Janmaleki M., Pachenari M., Seyedpour S.M., Shahghadami R., Sanati-Nezhad A. (2016). Impact of Simulated Microgravity on Cytoskeleton and Viscoelastic Properties of Endothelial Cell. Sci. Rep..

[71] Basirun C., Ferlazzo M.L., Howell N.R., Liu G.J., Middleton R.J., Martinac B., Narayanan S.A., Poole K., Gentile C., Chou J. (2021). Microgravity × Radiation: A Space Mechanobiology Approach Toward Cardiovascular Function and Disease. Front. Cell Dev. Biol..

[72] Topal U., Zamur C. (2021). Microgravity, Stem Cells, and Cancer: A New Hope for Cancer Treatment. Stem Cells Int..

[73] Infanger M., Kossmehl P., Shakibaei M., Baatout S., Witzing A., Grosse J., Bauer J., Cogoli A., Faramarzi S., Derradji H. (2006). Induction of three-dimensional assembly and increase in apoptosis of human endothelial cells by simulated microgravity: Impact of vascular endothelial growth factor. Apoptosis.

[74] Bavi N., Richardson J., Heu C., Martinac B., Poole K. (2019). PIEZO1-Mediated Currents Are Modulated by Substrate Mechanics. ACS Nano.

[75] ElGindi M., Sapudom J., Ibrahim I.H., Al-Sayegh M., Chen W., Garcia-Sabaté A., Teo J.C.M. (2021). May the Force Be with You (Or Not): The Immune System under Microgravity. Cells.

[76] Cazzaniga A., Ille F., Wuest S., Haack C., Koller A., Giger-Lange C., Zocchi M., Egli M., Castiglioni S., Maier J.A. (2020). Scalable Microgravity Simulator Used for Long-Term Musculoskeletal Cells and Tissue Engineering. Int. J. Mol. Sci..

[77] Davies J.E., Lopresto D., Apta B.H.R., Lin Z., Ma W., Harper M.T. (2019). Using Yoda-1 to mimic laminar flow in vitro: A tool to simplify drug testing. Biochem. Pharmacol..

[78] Caputo A., Caci E., Ferrera L., Pedemonte N., Barsanti C., Sondo E., Pfeffer U., Ravazzolo R., Zegarra-Moran O., Galietta L.J. (2008). TMEM16A, a membrane protein associated with calcium-dependent chloride channel activity. Science.

[79] Llanos P., Contreras-Ferrat A., Georgiev T., Osorio-Fuentealba C., Espinosa A., Hidalgo J., Hidalgo C., Jaimovich E. (2015). The cholesterol-lowering agent methyl-β-cyclodextrin promotes glucose uptake via GLUT4 in adult muscle fibers and reduces insulin resistance in obese mice. Am. J. Physiol. Endocrinol. Metab..

[80] Shi J., Hyman A.J., De Vecchis D., Chong J., Lichtenstein L., Futers T.S., Rouahi M., Salvayre A.N., Auge N., Kalli A.C. (2020). Sphingomyelinase Disables Inactivation in Endogenous PIEZO1 Channels. Cell Rep..

